# Milk

**Published:** 1878-10

**Authors:** 


### MILK.

Many persons imagine that the milk of cows is one of the most healthful of all articles, and yet it is a great mistake, except under certain limitations. By stout, strong, hardy, industrious out-door working men it may be used advantageously for breakfast and dinner, but, except in tea and coffee, and now and then half a glass for breakfast or dinner, it is not a proper article of food for invalids. In many instances patients have said to me: "I used to be a dear lover of milk, but I thought it made me bilious, and I have ceased using it altogether." This is the common-sense observation of ordinary men—one that, without any theory, and against a lifetime of prejudice, has forced itself upon the attention.

The rule that a man may eat almost anything with impunity, applies to one in good health, eating in moderation, according to the quality of the food; but when an invalid is to be fed, very different principles are to govern.

In all that I may say, I ask credence for nothing, except in proportion as it is followed up by the argument of *whole facts.*

